# The effectiveness of sound-based interventions on promoting sleep quality in hospitalized patients: A systematic review

**DOI:** 10.1016/j.isci.2026.117698

**Published:** 2026-09-22

**Authors:** Helga Rafael Henriques, Márcia Pereira Silva, Rui Guerreiro, Sílvia Caldeira Mendonça, Cândida Durão, Maria Rosario Pinto, Joana Teixeira

**Affiliations:** 1 Universidade de Lisboa, Escola Superior de Enfermagem da Universidade de Lisboa, Lisbon Center for Research, Innovation, and Development in Nursing (CIDNUR-ESEUL), Av. Prof. Egas Moniz, 1600-096 Lisbon, Portugal; 2 Unidade Local de Saúde Gaia e Espinho, R. Conceição Fernandes, 4434-502 Vila Nova de Gaia, Portugal; 3 Escola Superior de Saúde Do Instituto Politécnico de Santarém, Quinta do Mergulhão, Srª da Guia, 2005-075 Santarém, Portugal; 4 Unidade Local de Saúde Do Arco Ribeirinho, Barreiro Montijo Hospitalar Center, R. Machado Dos Santos 54, 2870-351 Montijo, Portugal

**Keywords:** environmental health, inpatients, nursing care, sleep quality, sound

## Abstract

Sleep quality strongly affects hospitalized patients’ recovery, yet pain, anxiety, and environmental factors such as noise and light frequently disrupt it. Sound-based therapies offer a promising, non-pharmacological approach, but their effectiveness has not been comprehensively synthesized. This systematic review examined fifteen studies (2012–2026, 2024 patients, mostly intensive care settings) evaluating sound-based interventions, including calming music, nature sounds, and white noise, on sleep quality. Intervention duration and timing varied widely, from brief five-minute sessions to continuous overnight administration, and sleep quality was most often measured using validated questionnaires. Despite substantial heterogeneity, sound-based interventions were associated with an overall improvement in sleep quality, although effect sizes varied considerably and several studies showed no significant benefit. These findings support the potential of low-cost, non-pharmacological sound interventions to improve sleep in hospital settings, while underscoring the need for further research to clarify which intervention characteristics drive effectiveness.

## Highlights

• Sound-based interventions improved sleep quality in hospitalized adults
• Benefits persisted after excluding the lowest-quality study
• Randomized and quasi-experimental studies showed consistent benefits
• Effects varied across sound types, settings, and delivery methods

## Graphical abstract

**Figure:**
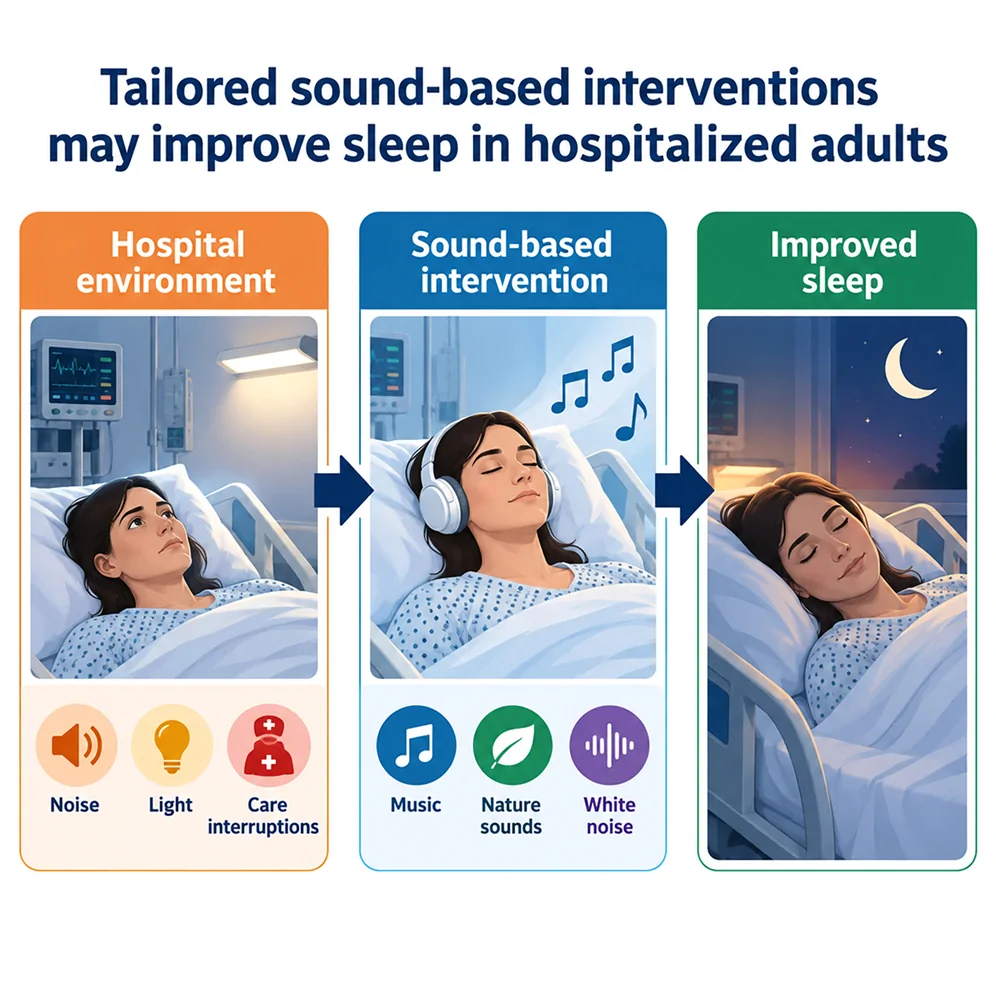

## Teaser

Biomedical discipline; Physiological state

## Introduction

Sleep involves a complex interplay of psychological, electrophysiological, neurochemical, endocrinological, immunological, and genetic factors. It is crucial for maintaining and restoring mental and physical well-being.^1^

Sleep quality is paramount in determining hospitalized patients’ overall well-being and recovery.^2^^,^^3^^,^^4^ Various factors, such as anxiety, pain, fever, and illness, can disrupt sleep. In addition, the hospital environment, characterized by continuous noise, light, and clinical activities, poses significant challenges to achieving restful sleep.^5^^,^^6^ In hospital settings, patients grapple with suboptimal sleep quality.^7^^,^^8^^,^^9^ The adverse effects of sleep loss are cardiovascular dysfunction, cognitive disturbance (delirium), psychomotor impairment (falls), psychological disturbance (anxiety), increased pain perception, metabolic disorder, catabolic propensity, immune dysfunction, and proinflammation.^2^ Sleep disturbance can affect patients up to 12 months after hospital discharge.^10^

Interest in environmental control over sleep-disrupting noise in healthcare settings dates back to Nightingale’s^11^ foundational writings on the therapeutic hospital environment. Systematic investigation of auditory interventions specifically emerged decades later: early experimental work established that white noise could mask disruptive environmental sounds and facilitate sleep onset in non-hospitalized populations, and by the early 1990s, randomized trials had begun testing recorded nature sounds in hospitalized adult populations; for example, Williamson^12^ reported that ocean sounds improved postoperative sleep in cardiac surgery patients, a study identified during our search but for which full-text access could not be obtained. Recognition of hospital noise as a modifiable, quantifiable problem was later formalized by international guidelines recommending maximum permissible sound levels in patient care areas.^13^ Since the early 2010s, a growing body of randomized and quasi-experimental trials has tested increasingly diverse sound-based approaches, including classical and relaxation music, white noise, and recorded nature sounds, across intensive care and general ward settings, reflecting both growing clinical interest and the continued absence of standardized implementation guidance.

Several prior reviews have examined components of this question, but each leaves an important gap. Bion et al.^14^ provided a narrative (non-systematic) review of sound and light reduction strategies limited to the adult ICU. Kakar et al.^15^ and Jespersen et al.^16^ each meta-analyzed music interventions specifically, without addressing white noise or nature sounds, and largely excluded general ward populations. Fang et al.^17^ conducted a network meta-analysis of combined sound-and-darkness interventions restricted to critically ill patients, without isolating the specific contribution of auditory stimuli. Most recently, Mendonça et al.^18^ reviewed nursing interventions for sleep in hospital wards broadly, without a specific focus on sound-based approaches. Collectively, no existing synthesis has quantitatively compared the effectiveness of different types of sound-based interventions—music, white noise, and nature sounds—across both intensive care and general ward settings, nor examined how delivery format (pre-recorded versus live/interactive) may moderate their effectiveness. This fragmentation limits the ability of nurses and other clinicians to select and implement the most appropriate sound-based strategy for a given clinical context. Addressing this gap is important because poor sleep in hospitalized patients is independently associated with delirium, prolonged recovery, and impaired quality of life, and because sound-based interventions are low-cost, non-pharmacological, and broadly implementable by nursing staff without specialized equipment. This is consistent with existing evidence that nurse-led interventions can positively impact patients’ perceived sleep quality in acute and semi-acute units,^19^^,^^20^ and further supports nurses’ capacity to implement sound-based strategies as part of routine care. This systematic review therefore aims to comprehensively evaluate and compare the effect of different types of sound-based interventions on sleep quality across hospitalized adult populations, synthesizing evidence across both intensive care and general ward settings.

### Methods

A systematic literature review was conducted using a structured and transparent approach, guided by the PICO (Participant, Intervention, Comparison, and Outcome) framework, to assess the impact of sound-based interventions on sleep quality in hospitalized patients.^21^ This review protocol was registered in the International Prospective Register of Systematic Reviews (PROSPERO) (CRD42024511972).

#### Eligibility criteria

Our research question was framed in a PI(C)O format (Population (P)/Intervention (I)/Comparison (C)/Outcome (O)): What is the effect of a sound-based intervention (I) on promoting the sleep quality (O) of hospitalized adults (P)?

We included studies involving patients who stayed overnight at the hospital. Sound-based interventions were eligible for inclusion regardless of delivery format, encompassing pre-recorded auditory stimuli (e.g., music, white noise, nature sounds) as well as live or interactively delivered music therapy administered by a trained professional (e.g., a music therapist). We incorporated studies facilitating comparisons across various groups, encompassing interventional studies (randomized or before-and-after) and observational studies (cohort and case-control), focusing on hospitalization. Hence, cross-sectional studies were excluded due to their inability to establish the temporal correlation between the outcome (sleep quality) and exposure (sound-based intervention).^22^ This limitation is especially concerning given the multifactorial nature of sleep health,^23^ particularly within hospital settings. Following Kesmodel,^22^ caution is warranted when drawing causal inferences from cross-sectional data unless the exposure can be reasonably presumed to remain stable over time.

Studies involving children, pregnant women, or women in labor were excluded. Emergency patients and outpatients were also excluded. In addition, systematic reviews, commentaries, editorials, and conference abstracts were not eligible for inclusion.

#### Search strategy

A comprehensive search was conducted across five online databases: Medical Literature Analysis and Retrieval System Online (MEDLINE), Cumulative Index to Nursing and Allied Health Literature (CINAHL), Cochrane Database of Systematic Reviews, Web of Science, and Scopus. The list of bibliographic references from the literature reviews identified on the topic was also reviewed. No temporal limit was imposed on the search. The search was conducted in July 2026, focusing on titles and abstracts, and performed by two researchers (HRH and JT). The following strategy was employed: (“critically ill patient” OR “critical care” OR intensive care units OR “acute patient” OR hospital∗ OR ward) AND (sleep OR “sleep quality” OR “sleep duration” OR “sleep hygiene”) AND (sound OR music OR “therapeutic sounds”). Data were limited to materials published in Portuguese, English, and Spanish.

#### Outcome

The primary outcome evaluated was the sleep quality of hospitalized individuals. Sleep quality is a multifaceted construct, encapsulating latency to sleep onset, sleep duration, sleep depth, and subjective sleep satisfaction upon awakening.^19^ For an assessment to be recognized as a valid measure of sleep quality, it must be quantified using psychometric instruments that are both valid and reliable, or verified against predetermined threshold values that indicate poor or satisfactory sleep quality.

#### Selection process

The references found were exported to the web-based platform Rayyan QCRI. The research team members (HRH, JT, RG, and MS) worked on the screening process. Using the Rayyan QCRI deduplication system, similarity records were manually checked and removed if they were duplicates. After removing duplicate studies, the team independently reviewed the titles and abstracts to identify potentially relevant studies, ensuring that at least two reviewers reviewed the entire sample. A third reviewer (HRH) intervened to seek consensus in case of discrepancies. The full-text articles were then thoroughly reviewed in detail against the eligibility criteria. The librarian was involved in searching for articles that needed to be fully available. Whenever we were unable to access the full article, the author was contacted. The team resolved any discrepancies to ensure consensus. The research team used a standardized Excel spreadsheet to extract information from the studies. To streamline the team’s efforts, this form was seamlessly accessible in real-time and served as a shared online platform, ensuring all members of the data extraction team could collaborate efficiently.

#### Data collection process and data items

All research team members collected the data using a standardized form that had been previously developed and tested to ensure consistency and clarity throughout the extraction process. For each included study, data were extracted on study identification (authors, year, and journal), the country where the study was conducted, study type, level of evidence, study aim, characteristics of the participants, details of the intervention, type of sound used, sleep-related outcomes, main conclusions, and reported study limitations.

In addition to these core elements, further variables were collected to ensure a comprehensive characterization of the studies. These included: participants’ demographic information (such as age, gender, and health status, when available); intervention details (including timing, duration, frequency, and the professional responsible for administering the intervention); type of auditory stimulus (e.g., classical or relaxation music, white noise, nature sounds, traditional music, and live/therapist-administered music therapy); sleep outcome measures (specifying whether the focus was sleep quality or sleep duration, and identifying the instruments used for assessment); and funding sources, where disclosed.

Unavailable data were recorded as “not reported” and excluded from any synthesis that required that specific information. In cases of ambiguity—particularly concerning intervention protocols or outcome measures—interpretations were based on the methodological descriptions provided by the authors. When essential data were lacking, study authors were contacted for clarification. If no response was obtained within four weeks, the study remained included in the review, and the analysis proceeded based on the available information.

This structured and transparent method for data collection and assumption management aimed to ensure rigor and comparability across studies, supporting the validity of subsequent synthesis and interpretation.

#### Study risk of bias assessment

Each study was independently assessed using the Critical Appraisal Checklist Tools proposed by the Joanna Briggs Institute. Discrepancies in assessing the risk of bias were resolved through discussions between the two reviewers (HRH, JT), with a third author participating as needed to ensure the quality of the appraisal process. All assessments were conducted independently, and no automation tools were used in the risk of bias assessment. The process was fully manual to ensure careful consideration of study context and methodological details.

Risk-of-bias ratings were used to characterize study quality and contextualize confidence in individual findings, but were not used to exclude studies or to weight the pooled meta-analysis.

#### Data synthesis

We utilized the standardized mean difference (SMD) as the primary effect measure for the outcome of sleep quality, given the diversity of validated instruments used across studies (e.g., RCSQ, PSQI, VSH). This approach enabled the comparison of results on a common scale and provided a comprehensive estimate of the overall effect of sound interventions. Given the high anticipated clinical and methodological heterogeneity across studies–including differences in clinical setting, sound delivery format (pre-recorded versus live/interactive), and outcome instruments—a random-effects model (DerSimonian-Laird) was used to pool the standardized mean difference,^24^ rather than a fixed-effect model, which assumes a single true underlying effect. This decision was confirmed post hoc by the observed heterogeneity (*I*^2^ = 97%), classified as highly severe by the pre-specified thresholds above. Sleep duration outcomes were reported narratively rather than pooled, as an insufficient number of studies provided data suitable for quantitative synthesis. Studies with a crossover design lacking an independent control arm^25^ were described narratively but not included in the pooled meta-analysis, as their data violate the independent-groups assumption of the inverse-variance model. A sensitivity analysis was pre-specified to assess the influence of study quality on the pooled estimate, excluding studies rated below 60% on the JBI critical appraisal checklist.

Quasi-experimental studies were defined as those employing an intervention and comparison group without random allocation to conditions (e.g., non-randomized controlled trials or before-and-after designs with a comparison group). Given the lower internal validity typically associated with the absence of randomization, we conducted a pre-specified subgroup analysis comparing pooled estimates for randomized controlled trials (RCTs) versus quasi-experimental studies.

The I^2^ statistic was utilized to quantify the variability in effect estimates attributable to heterogeneity rather than chance. Heterogeneity was categorized as mild (I^2^ < 25%), moderate (*I*^2^ = 25–50%), severe (*I*^2^ = 50–75%), or highly severe (*I*^2^ > 75%). Additionally, the *Z* test was calculated to determine the statistical significance of the overall treatment effect.^21^

The results of the meta-analyses were graphically represented using forest plots, which illustrated point estimates along with 95% confidence intervals. Given the substantial heterogeneity observed, data synthesis was complemented by a descriptive analysis, guided by the Synthesis Without Meta-analysis (SWiM) guideline,^26^ while also adhering to the principles of the Preferred Reporting Items for Systematic Reviews and Meta-Analyses (PRISMA) guideline.^27^ A narrative approach was employed to organize and interpret the evidence within each domain of interest.

## Results

### Study selection

Electronic database searches collected 1524 articles. Duplicate removal identified 726 articles for screening, which were analyzed by title and abstract, excluding 673 papers based on the established inclusion criteria. At this stage, 53 articles were for full-text review and eligibility assessment, of which 8 were not retrieved in full text. The remaining 12 studies were included in this review.

Within the excluded study sample, 4 reviews on the topic were identified.^15^^,^^28^^,^^29^^,^^30^ Their lists of bibliographical references underwent manual analysis, leading to the inclusion of three additional studies.

The final sample had 15 articles. This process is outlined in the PRISMA flowchart (Figure 1).

**Figure 1:**
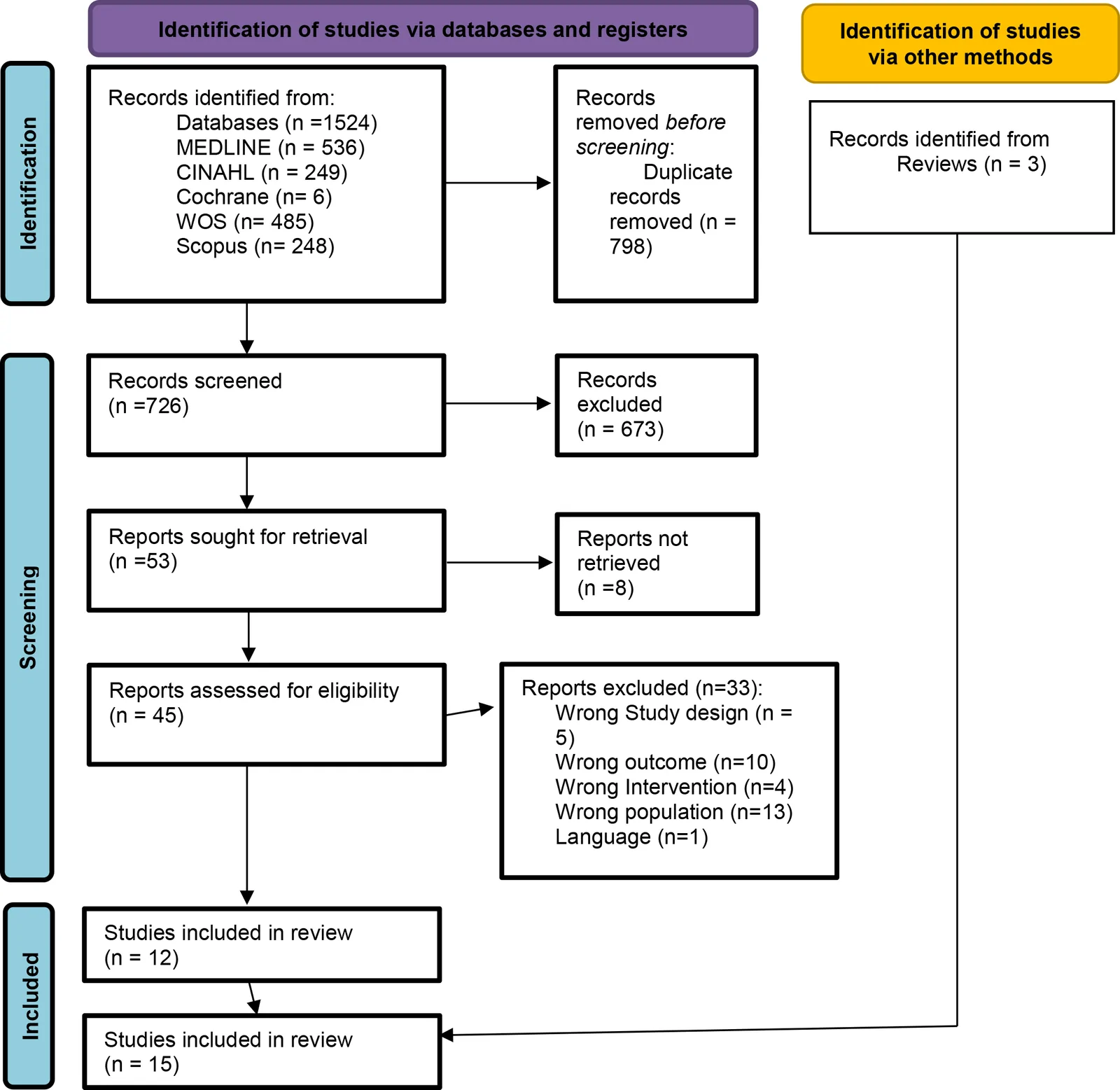
PRISMA flowchart

### Studies characteristics

The included studies were published between 2012 and 2026. Among the fifteen final studies obtained, ten are randomized controlled trials (RCTs), and five are quasi-experimental studies. A total of 2024 participants were enrolled/randomized across the 15 included studies. After accounting for withdrawals in two studies (Afshar et al.^31^, *n* = 2; Chaudhary et al.^25^, *n* = 8), 2024 participants were included in the final analyses. Sample sizes ranged from 28^32^ to 931^33^ participants per study. Except for four studies,^33^^,^^34^^,^^35^^,^^36^ which were conducted in a cardiac rehabilitation setting or a general surgical ward rather than an ICU, all others were conducted in intensive care units (ICU). Most of the studies took place in ICU settings.^25^^,^^31^^,^^32^^,^^37^^,^^38^^,^^39^^,^^40^^,^^41^^,^^42^^,^^43^^,^^44^ The results from these studies are organized and summarized in Table 1, shown later in discussion.

**Table 1:** Sound-based interventions for sleep promotion

Authors	Country	Study type	Evidence level	Aim	Participants	Interventions	Sound type	Sleep measure	Conclusions	Limitations of the study
Ryu et al.^42^	Korea	RCT	Level 1.c	compare the effect of earplug-delivered sleep-inducing music on sleep in persons with percutaneous transluminal coronary angiography (PTCA) in the cardiac care unit.	58 participants (control group: 29 and intervention group: 29)	earplug-delivered sleep-inducing music and eye shield versus no music (but earplugs and eye shield). If a subject fell asleep with the music still in progress, the earphone was not removed intentionally until 5 a.m., so as not to disturb sleep.	music (nature sounds, delta wave control music, Goldberg variations).	quantity of Sleep questionnaire (self-reported total sleeping minutes, calculated from time of falling asleep to time of awakening) and the Modified Verran and Snyder-Halpern (VSH) sleeping scale (8 items, Likert 0–10, total score 0–80).	sleeping quantity and quality were significantly higher in the experimental group than in the control group, due to the earplugs and sleep-inducing music.	this study tested the quality and quality of sleep only one time. To increase the validity and reliability of the research findings, testing at different moments is needed.
Su et al.^32^	Taiwan	RCT	Level 1.c	to examine the effects of non-commercial music on quality of sleep and relaxation indices, including heart rate, mean arterial pressure, and respiratory rate in patients in intensive care units.	28 patients - intervention group: 14; control group: 14.	interventions consisted of four pieces of sedating piano music (45 min total), composed by two of the study authors, played on a CD player continuously until finished. The pieces shared common characteristics: smooth rhythm, tempo of 60–80 beats/min, minor tonalities, smooth melody lines, and no dramatic changes in volume or rhythm; volume was set at 30–40 dB. Music was played once, scheduled in the evening between 21:30–23:30.	sedating piano music	Verran & Snyder-Halpern (VSH) Sleep Scale (subjective, administered before and after intervention) and polysomnography (PSG; objective, first 2 h of nocturnal sleep).	findings suggest that patients in medical ICU may benefit from improved stability of vital signs and quality of sleep if nurses offer a short period for listening to music at nocturnal sleep time.	there might be a very large number of potential confounding variables of sleep in ICU patients and the heterogeneity of the participants.
Hu et al.^38^	China	RCT	Level 1.c	to determine the effects of using earplugs and eye masks with relaxing background music on sleep, melatonin and cortisol levels in ICU patients.	45 patients (20 in the intervention group, 25 in the control group)	sounds of nature and bird songs were selected to imply morning. Sounds of frogs and waves were selected to imply evening. Pieces of classical music were selected as relaxing music. Patients used earphones to listen to the corresponding music at 8:00 to 9:00 p.m. and 7:30 to 8:30 a.m. every day after surgery. The duration was 30 min.	Classical music (e.g., Blue Danube, Moonlight Sonata, Lofty Mountains and Flowing Water, Clouds Chasing the Moon, Lotus Emerging out of Water, among others) and nature sounds (bird songs to imply morning; frog and wave sounds to imply evening).	Chinese version of the Richards-Campbell Sleep Questionnaire (RCSQ; 6-item visual analog scale, 0–100 mm) as the primary subjective sleep outcome. The Pittsburgh Sleep Quality Index (PSQI) was used only at baseline, before surgery, to characterize participants’ usual sleep quality. Urinary melatonin (6-SMT) and cortisol were also measured as objective biomarkers.	the findings provided evidence for nurses to use soothing music as a research-based nursing intervention for intensive care unit patients’ sleep improvement.	the study only evaluated subjective sleep quality and did not carry out an objective sleep assessment. The study only assessed a 12-h nocturnal period rather than over 24 h during the first two nights in the ICU. This study included a small and specific population in a CSICU (results may not be applicable to all settings and patients).
Afshar et al.^31^	Iran	quasi-experimental study	level 2.d	to determine the effect of white noise on the quality of sleep in patients admitted to the ICU	62 patients enrolled and randomized to two parallel groups (control *n* = 31; experimental *n* = 31); 2 patients withdrawn (1 per group), leaving 30 per group for analysis. Both groups were assessed pre-intervention (first day of admission) and again after three nights (control: no intervention; experimental: white noise).	white noise broadcast at an intensity of 40–50 dB (reported as 50–60 dB in the article’s abstract; Methods specify 40–50 dB) for 1 h per night, on three consecutive nights, during pre-identified noisy periods of the ward (8:00–9:00 pm and 11:00 pm–12:00 am).	white noise	PSQI	the use of white noise is recommended as a method for masking environmental noises, improving sleep, and maintaining sleep in the coronary care unit.	limitations of the study include the lack of isolated units, the inability to control all medications that affect sleep, and the short time spent examining sleep. The researchers recommend that future studies select a larger sample size and examine the effect of continuous white noise application all night.
Hansen et al.^37^	Denmark	RCT	level 1.c	to investigate the effects of music on self-reported quality of sleep during daytime rest among patients in the intensive care unit.	37 patients (19 in the control group and 18 in the intervention group)	participants in the intervention group listened to music for 30 min during a single daytime rest period in the ICU (one session only; not administered nocturnally). The music, “MusiCure”, was specially composed for critically ill ICU patients by Niels Eje and consisted of four pieces of soothing music (soft wind, bird twitter, ocean sound and musical instruments). Unlike headphone-delivered interventions, the music was played through a loudspeaker placed in the ceiling above the participant’s bed (Soundfocus method), centring the sound around the patient. Volume was set at a comfortable level for each participant. The control group rested without music (standard care).	“MusiCure” (composed by Niels Eje): 4 pieces combining nature sounds (soft wind, bird twitter, ocean sound) and musical instruments; categorised as “music medicine” (passive, pre-recorded, not therapist-administered).	Danish version of the Richards-Campbell Sleep Questionnaire (RCSQ), a 5-item visual analog scale (0–100 mm: depth, latency, awakenings, time awake, overall quality); the Danish version adds an optional 6th item on perceived nocturnal noise level. Administered once, after the daytime rest period (not validated for this daytime-rest use, per the authors).	listening to music during daytime rest improves subjective sleep in patients in the ICU. Furthermore, some indications listening to music reduces the subjective experience of noise in some patients. The result of this study implies that music can be an effective practice for nurses to improve sleep.	the study only measures the subjective quality of sleep and the perceived noise level and does not incorporate objective measurements. Intervention and statistical analyses were not blinded, which impacts the validity. The subjective quality of sleep was only measured once. The RCSQ is developed to measure the nocturnal quality of sleep and in this study, it is used to measure the quality of sleep during daytime.
Pagnucci et al.^41^	Italy	quasi-experimental study	level 2.d	to identify if complementary interventions impacted conscious intensive care patients’ perception of stress factors and quality of sleep.	74 patients pre- and post-intervention	two simultaneous treatments were administered on the second ICU night: (1) receptive musical sounds–each patient chose either their own preferred music from a digital library or relaxing nature sounds, played via MP3 player through headphones, left playing for 7 h (11 pm–7 am); and (2) a feet and leg massage using sweet almond oil with added lavender and lemon aromas, lasting 20 min, performed by a nurse trained in the technique and assisted by a student nurse. No patient requested the music to be stopped.	patient-selected music (digital library) or relaxing nature sounds, played via MP3 player and headphones.	modified Richards-Campbell Sleep Questionnaire (6 items: sleep depth, latency, awakenings, returning to sleep, sleep quality, noise; colored analog scale 0–100 per item; bands: 1–20 very poor, 21–40 poor, 41–60 acceptable, 61–80 good, 81–100 very good sleep).	a multi-pronged approach could improve the results attained even further and greatly aid ICU patients in getting a good night’s sleep.	the study included mostly surgical patients and no intubated patients were included in the study limiting the generalizability of the results to ICUs. There are factors that may influence the quality of sleep perceptions. Thus, a randomized controlled trial is needed to reduce bias.
Chaudhary et al.^25^	India	RCT	level 1.c	assess and compare the effectiveness of earplugs and eye masks versus ocean sound on sleep quality among ICU patients.	68 patients randomized (crossover design, no separate control/intervention groups – participants acted as their own control): Group 1 (*n* = 34) and Group 2 (*n* = 34), each receiving both interventions in reverse order across 4 nights (1st night: baseline/no intervention; 2ND night: Group 1 = earplugs+eye mask, Group 2 = ocean sound; 3rd night: washout/no intervention; 4th night: crossed over). 8 withdrawals (transferred/discharged); 30 analyzed per group (*N* = 60 total).	nocturnal nine-hour period (10:00 pm–7:00 am) assessed over 4 nights. Earplugs and eye mask: foam earplugs (3M; noise reduction rating 29 dB, single number rating 37 dB) plus a dark cloth eye mask (18.5 × 8.5 cm), worn 10:00 pm–7:00 am (9 h). Ocean sound: a type of white noise (soothing ocean-wave sound), delivered via earphones and an Android app (“Relax Melodies Meditation”, Ipnos software) for 30 min at sleep onset, from 9:15–9:45 pm (i.e., before the 9-h window, not throughout it). Interventions were crossed over between the two groups (see Participants column).	ocean sound (a type of white noise, soothing ocean-wave sound) delivered via the “Relax Melodies Meditation” Android app (Ipnos software), compared against earplugs (3M foam) plus an opaque eye mask.	a structured 11-item self-reported sleep quality scale, developed by the researchers by modifying the PSQI (which patients found difficult to use) and drawing on the RCSQ and the Verran & Snyder-Halpern scale. Items: sleep quality, depth, latency, awakening, returning to sleep, noise, light, duration, status of freshness, disturbance of daily activities, and sleep efficiency. Each item is scored from 0 (very good) to 3 (very bad); total score range 0–34, higher = worse sleep. Content validity index (S-CVI) = 0.87; reliability = 0.85.	the majority (76.6%) of the participants reported the presence of sleep disturbance in the ICU. Earplugs and eye masks were better than ocean sound in improving sleep quality.	the variation in outcome might be because the eye mask and earplugs are two simultaneous interventions, whereas ocean sound was a single intervention. Secondly, the duration of the intervention’s implementation is different. The sample size was small and in a single setting.
Kim et al.^40^	Korea	RCT	level 1.c	to investigate the effects of patient-directed interactive music on saliva melatonin levels and sleep quality among postoperative elderly patients in the intensive care unit (ICU).	133 elderly patients were randomized into three groups (1 control group with 45 patients, and 44 + 44 patients for the other 2 groups.	three groups: (1) Interactive Music Therapy (IMT, *n* = 44): a daytime session of 15–20 min with a certified music therapist (breathing exercise with music → interactive music-making matched to patient’s preferred genre → repeat breathing exercise) PLUS 30 min of individual music-listening at night (8–10 pm) via MP3 player and earphones; (2) Passive Listening (PL, *n* = 44): night-time-only 30-min listening to therapist-preselected relaxing music (patient could reject a list and be offered another), MP3 player auto-stopped after 30 min; (3) Control (*n* = 45): routine care only, no music. Both music groups’ songs were based on patient preference where possible.	for the Passive Listening group specifically: pre-selected classical or contemporary instrumental pieces (e.g., Air for G string, Allemande, Canon, Nocturne, Swan). The IMT group’s music was individualized to each patient’s self-reported preferred genre, not restricted to classical/instrumental.	Richards-Campbell Sleep Questionnaire (RCSQ; 5-item VAS, 0–100 mm), administered preoperatively and again on postoperative days (POD) 1 and 2; the primary outcome was the POD2 score. Secondary outcomes included saliva melatonin and cortisol (measured at three time points) and the Quality of Recovery-40 (QoR-40) questionnaire.	single interactive music therapy intervention improved subjectively assessed short-term sleep quality in postoperative elderly patients	small study population. Limited to patients who are capable of spontaneous ventilation and are relatively less severe. RCSQ is a subjective questionnaire. The short-term effects were evaluated in only one music intervention session.
Herscher et al.^33^	USA	quasi-experimental study	Level 2.d	improve sleep quality among hospitalized patients as measured by the Richards- Campbell Sleep Questionnaire (RCSQ).	931 patients (pre- and post-intervention groups were the same).	relaxing music was played overhead in the hallway for approximately 5 min at 9:00 p.m. with other measures (eye mask, earplugs, lavender scent pad, and non-caffeinated tea bags).	relaxing music	quality by RCSQ	overall improvement in self-reported sleep with a validated tool. Further studies are needed to determine whether the effects demonstrated in this project produce corresponding benefits on clinical outcomes (for example: delirium and length of stay).	the focus of the project was a multimodal intervention. This limits the ability to determine which aspects of the bundle might have been more or less effective at sleep promotion. This was a single-center study, and it is unknown if it can be generalized.
Duru et al.^34^	2022 Turkey	Quasi-experimental study	Level 2.d	evaluate the effect of music-assisted progressive relaxation exercises (MaPRE) on postoperative sleep quality and pain intensity of patients hospitalized in the surgical clinic.	62 patients in a non-randomized, single-blind, quasi-experimental controlled design: 31 in an intervention group (performed MaPRE regularly) and 31 in a separate, non-randomized control group (did not perform MaPRE); both groups assessed pre- and post-intervention (i.e., two distinct parallel groups, not a single pre/post group).	the intervention group regularly performed MaPRE for an average of 30 min per day, before sleep, for one week after surgery. MaPRE combined pre-recorded relaxation music (prepared by the Turkish Psychological Association, installed on the patient’s own mobile phone) with Jacobson’s Progressive Relaxation Exercises (conscious contraction/relaxation of muscle groups with deep breathing, performed sitting or lying comfortably, accompanied by the music). Nursing students (who completed a 4-h training program) taught MaPRE to each patient at the bedside using an illustrated brochure and verified correct technique; adherence was checked via patient self-report and nurse observation. The control group received no MaPRE.	pre-recorded relaxation music prepared by the Turkish Psychological Association (specific tracks/composition not reported by the authors); played on the patient’s mobile phone during the relaxation exercise.	visual Analog Sleep Scale (VASS) – Turkish version of the Verran & Snyder-Halpern scale, adapted to 10 items (without the original’s 3 sub-dimensions); each item scored 0–100 by visual analog technique, total score range 0–1000; higher score = worse sleep quality. Cronbach’s alpha = 0.87 (pre- and post-test) in this study.	MaPRE increases the sleep quality of patients in surgical clinics. MaPRE is a non-invasive, affordable, effective, and easy-to-apply intervention that can be applied throughout hospitalization.	there was no randomization so that all patients could benefit from the advantage of the intervention.
Sayed et al.^43^	Egypt	quasi-experimental study	Level 2.d	to investigate the effectiveness of alternative methods to nursing strategies in promoting good night sleep patterns among coronary intensive care patients.	85 conscious patients (pre and post-intervention)	administered on a single night only (the second ICU night; the first night served as an unexposed baseline). Patients listened to self-selected calming/relaxing music sounds via a digital MP3 player and headphones. Simultaneously with the music, a researcher-administered shoulder massage using almond oil with added lavender and lemon aromas was given for 15 min. The music continued playing for the remainder of the night after the massage ended (i.e., massage+aromatherapy = 15 min concurrent with the start of music listening; music alone = rest of the night). Sleep quality and stress were reassessed the following (third) day.	slow rhythm, simple repetitive tempos; patient self-selected/preferred music – the authors do not report a specific playlist or musical composition.	Richards-Campbell Sleep Questionnaire (RCSQ; 6 components – sleep depth, latency, awakening, returning to sleep, sleep quality, noise; colored analogue scale 0–100 per item; bands: 1–20 very poor, 21–40 poor, 41–60 well-accepted, 61–80 good, 81–100 very good), embedded within a broader “quality of sleep sheet” that also collected bio-demographic data. A separate Stress Factors in the ICU Questionnaire (SEDAICU; 32 items across 4 domains: environment, feelings, emotions, physical state) was used as a secondary/co-measure of perceived stress.	alternative nursing strategies improved the sleep quality of ICU patients using aromatherapy, body massage, listening to calm music, and decreased of stress factors in the ICU.	the focus of the study was a multimodal intervention. This limits the ability to determine which aspects of the bundle might have been more or less effective at sleep promotion. We do not know the effect of music only.
Kakar et al.^39^	Netherland	RCT	level 1.c	to investigate the effect of a music intervention on anxiety in adult critically ill patients admitted to the ICU	94 patients total (50 patients in the intervention group and 44 patients in the control group).	music was offered twice daily (morning session 09:00–12:00; evening session 20:00–23:00, before intended sleep) for at least 30 min per session, over 3 days, in addition to standard care; patients could request more/longer sessions. Delivered via over-the-ear headphones connected by Bluetooth to a tablet with pre-arranged online music lists based on the patient’s (or, if unable, legal representative’s/family’s) preference, assessed at baseline. Note: the study’s primary outcome was anxiety (VAS-A); sleep quality was a secondary outcome only.	highly individualized, patient-preference-based selection (assessed at baseline from a pre-arranged, rearrangeable list); rock and heavy metal were discouraged. Genre choice varied greatly; the most common genres reported were pop, Dutch-language music, and classical music.	visual numeric scale, 1–7 (1 = “did not/barely sleep”; 7 = “slept very well”); not a validated tool (acknowledged limitation by the authors). Assessed once daily as a secondary outcome; the study’s primary outcome was anxiety, measured separately with the visual analog scale for anxiety (VAS-A, 0–10).	on the first study day, patients in the control group reported a significantly higher quality of sleep than patients in the intervention group. On the remaining days, there are no statistically significant differences in sleep quality.	subjective outcome assessments have limitations in the ICU population since the medical condition and sedation may alter the patient’s responses. The intervention might have adversely influenced sleep quality, which was not assessed using a validated tool.
Yao et al.^35^	China	RCT	level 1.c	aimed to explore phase I cardiac rehabilitation combined with 5-phase music for the rehabilitation of acute myocardial infarction patients after emergency percutaneous coronary Intervention.	150 patients in three groups: control (50), cardiac rehabilitation (50), and rehabilitation-music groups (50)	five-phase music selection followed traditional Chinese medicine syndrome differentiation (e.g., “He Hua Ying Ri” for heart-yang insufficiency; “Bing Xue Han Tian” for kidney-yin deficiency); a combination of Zhi and Yu tunes (“Zi Zhu Diao”) was selected for participants with heart-fire exuberance. Music was delivered via individual MP3 players and earphones. Volume was set at 50–60 dB during the rehabilitation exercise session and 40–50 dB before sleep. During exercise, the corresponding track was played in a single cycle for 25–30 min (from warm-up through relaxation training); before sleep, the same 5-phase music was played in a repeated cycle for 30 min. The specific rhythm, tempo and duration-segmentation structure of the music composition itself are not reported by the authors.	five-phase music (Wu Xing/5-tone traditional Chinese music therapy, based on the tones Jue, Zhi, Gong, Yu and Shang, corresponding to the 5 elements, 5 Zang-organs and 5 types of qi); cassette composed by Shifeng Cong, produced by the Chinese Medical Multimedia Press. For this study: combination music of Zhi and Yu tunes (“Zi Zhu Diao”).	self-rating sleeping scaling (SRSS).	phase I cardiac rehabilitation combined with 5-phase music could alleviate anxiety and depression and improve sleep quality.	it was performed at a single hospital with a small sample size, limiting the generalization of the results.
Topal & Sürme.^44^	Türkiye	RCT	level I (RCT)	evaluate impact of eye masks and music on sleep quality and delirium in surgical ICU patients after abdominal surgery	*n* = 45 (21 intervention/24 control); adults, post-abdominal surgery, surgical ICU, RASS -1/0/+1, Nu-DESC<2 at baseline	eye mask + calming classical music (1h/night), applied together for 3 consecutive postoperative nights, vs. routine care	pre-recorded classical music (Chopin Nocturnes) COMBINED with eye mask (co-intervention, not sound-only)	Richard-Campbell Sleep Questionnaire (RCSQ, self-report)	music + eye mask significantly improved RCSQ scores (nights 2–3) and reduced delirium incidence/severity vs. control	single hospital; no blinding of patients/staff; quasi-random allocation (odd/even file numbers); short duration (3 days); cannot isolate independent effect of music from eye mask
Wen & Fan.^36^	China	randomized controlled trial (single-center)	Level I-II	evaluate effects of an IoT-based intelligent noise control system combined with personalized music therapy on postoperative pain, sleep quality, and emotional status after perianal abscess surgery	*n* = 162 (81 intervention/81 control); adults 18–65 years, first-episode perianal abscess, ASA I-II	IoT-based noise control (room modification, real-time monitoring, white-noise masking) PLUS personalized music therapy (2 × 30min/day) until discharge/day 5, vs. routine care	personalized pre-recorded music (patient-selected) COMBINED with environmental noise control/white-noise masking (co-intervention, not sound-only)	Richards-Campbell Sleep Questionnaire (RCSQ, self-report, nights 1–2)	combined intervention significantly improved RCSQ scores, reduced postoperative pain, and reduced anxiety/depression; cumulative benefit over time	bundled intervention - cannot separate contribution of noise control vs. music; no blinding possible; intervention/control rooms differed in size/type (confound); no objective sleep measures; single-center

### Qualitative appraisal

The outcomes of this systematic review underscore significant shortcomings in the methodological rigor of randomized controlled trials included in the critical appraisal analysis (Table 2). The absence of allocation concealment,^25^^,^^35^^,^^37^^,^^38^^,^^39^^,^^40^^,^^44^ participant blinding,^25^^,^^35^^,^^36^^,^^37^^,^^38^^,^^39^^,^^40^^,^^44^ blinding of treatment administrators,^25^^,^^35^^,^^36^^,^^37^^,^^38^^,^^39^^,^^44^ and blinding of outcome assessors^32^^,^^35^^,^^37^^,^^38^^,^^39^^,^^44^ further amplify the risk of bias. Collectively, these studies amplify the risk of bias. Therefore, it is imperative to exercise caution when interpreting the findings from these studies, considering the potential influence of these methodological inadequacies on the validity of the reported results.

**Table 2:** Qualitative appraisal

RCT		Was true randomization used for assignment of participants to treatment groups?	Was allocation to treatment groups concealed?	Were treatment groups similar at the baseline?	Were participants blind to treatment assignment?	Were those delivering the treatment blind to treatment assignment?	Were treatment groups treated identically other than the intervention of interest?	Were outcome assessors blind to treatment assignment?	Were outcomes measured in the same way for treatment groups?	Were outcomes measured in a reliable way	Was follow up complete and if not, were differences between groups in terms of their follow up adequately described and analyzed?	Were participants analyzed in the groups to which they were randomized?	Was appropriate statistical analysis used?	Was the trial design appropriate and any deviations from the standard RCT design (individual randomization, parallel groups) accounted for in the conduct and analysis of the trial?	
authors	year	1	2	3	4	5	6	7	8	9	10	11	12	13	
Ryu, Min-Jung and Park, Jeong Sook and Park, Heeok	2012	Y	N	Y	N	Y	N	Y	Y	Y	Y	Y	Y	Y	77%
Su, Chiu-Ping and Lai, Hui-Ling and Chang, En-Ting and Yiin, Lih-Ming and Perng, Shoa-Jen and Chen, Pin-Wen	2013	Y	N	Y	Y	Y	Y	N	Y	Y	N	Y	Y	Y	77%
Hu, Rong-Fang and Jiang, Xiao-Ying and Hegadoren, Kathleen M and Zhang, You-Hua	2015	Y	Y	Y	N	N	N	N	Y	Y	Y	Y	Y	Y	70%
Hansen, IP and Langhorn, L and Dreyer, P	2018	Y	N	y	N	N	y	N	y	Y	Y	Y	y	y	70%
Chaudhary, Abhilasha and Kumari, Vinay and Neetu, Neetu	2020	Y	N	N	N	N	Y	Y	Y	Y	N	Y	Y	Y	62%
Kim, Jeongmin and Choi, Dain and Yeo, Myung Sun and Yoo, Ga Eul and Kim, Soo Ji and Na, Sungwon	2020	Y	Y	Y	N	Y	N	Y	Y	Y	Y	Y	Y	Y	77%
Yao, Xu and Jin, Yi and Gao, Chunlan and Zhang, Yuqin and Lu, Yun and Li, Xiaoting and Ma, Lili	2023	Y	Y	Y	N	N	Y	N	Y	Y	Y	Y	Y	Y	77%
Kakar E, Ottens T, Stads S, Wesselius S, Gommers DAMPJ, Jeekel J, van der Jagt M.	2023	Y	N	Y	N	N	Y	U	Y	N	Y	Y	Y	Y	70%
Topal, Tuğçe and Sürme, Yeliz	2025	N	N	Y	N	N	Y	N	Y	Y	Y	Y	Y	Y	62%
Wen, Shuangshuang and Fan, Lisong	2026	Y	Y	Y	N	N	N	Y	Y	Y	Y	Y	Y	Y	77%

Quasi-experimental		Is it clear in the study what is the ‘cause’ and what is the ‘effect’ (i.e., there is no confusion about which variable comes first)?	Were the participants included in any comparisons similar?	Were the participants included in any comparisons receiving similar treatment/care, other than the exposure or intervention of interest?	Was there a control group?	Were there multiple measurements of the outcome both pre and post the intervention/exposure?	Was follow up complete and if not, were differences between groups in terms of their follow up adequately described and analyzed?	Were the outcomes of participants included in any comparisons measured in the same way?	Were outcomes measured in a reliable way?	Was appropriate statistical analysis used?	
authors	year	1	2	3	4	5	6	7	8	9	–
Afshar P, Bahramnezhad F, Asgari P, Shiri M.	2016	N	N	Y	N	N	Y	N	Y	Y	44%
Pagnucci, N., Tolotti, A., Cadorin, L., Valcarenghi, D., & Forfori, F.	2019	Y	y	y	y	y	y	y	y	y	100%
Herscher, Michael and Mikhaylov, Daniela and Barazani, Sharon and Sastow, Dahniel and Yeo, Ilhwan and Dunn, Andrew S. and Cho, Hyung J.	2021	Y	Y	N	N	Y	Y	Y	Y	Y	78%
Duru, Pınar; Örsal, Özlem; Şara, Yasemin; Köşgeroğlu, Nedime	2022	Y	Y	Y	Y	Y	N	Y	Y	Y	89%
Sayed, Z.A. and Ahmed, G.H. and Soliman, W.S.	2022	Y	N	Y	Y	Y	N	Y	Y	Y	78%

In the study by Kakar et al.,^39^ the outcomes were not reliably measured, as the research utilized a sleep evaluation scale that needed validation.

Afshar’s^31^ quasi-experimental study fails to establish a clear cause-and-effect relationship, lacks participant comparability across different groups, omits the inclusion of a control group, does not incorporate multiple measurements of outcomes pre- and post-intervention, and exhibits inconsistency in outcomes. These deficiencies compromise the study’s internal validity and reliability.

The JBI critical appraisal scores ranged from 44%^31^ to 100%^41^ among the quasi-experimental studies, and from 62%^25^^,^^44^ to 77%^32^^,^^35^^,^^36^^,^^40^^,^^42^ among the RCTs, confirming that methodological quality varied considerably across the included evidence base and was consistently lower for the non-randomized designs. To address the risk-of-bias concerns identified in Table 2, future trials in this field should prioritize central or third-party randomization and allocation concealment, the use of sham or attention-control auditory conditions (e.g., silent headphones or non-therapeutic ambient sound) to enable blinding of participants and intervention administrators, and independent, blinded outcome assessment using validated sleep instruments. Quasi-experimental studies such as Afshar et al.^31^ were retained in this review because they addressed an outcome (sleep duration) and intervention (white noise) that were otherwise underrepresented among the eligible randomized trials. Given its lower JBI appraisal score (44%) and quasi-experimental design, we consider the evidence from this study to be of lower certainty than that from the included RCTs; accordingly, a sensitivity analysis was conducted to assess its influence on the pooled estimate (see later in discussion). This distinction should be read as a methodological qualification rather than as an endorsement of equivalent evidentiary weight across study designs.

### Sound-based intervention

Different sound interventions were applied to the patients to promote sleep. The data indicate that the sleep-induced sounds highlighted in the literature included classical music,^32^^,^^36^^,^^37^^,^^38^^,^^39^^,^^40^^,^^42^^,^^44^ relaxation music,^33^^,^^34^^,^^42^^,^^43^ sounds of nature,^25^^,^^37^^,^^42^ white noise,^31^ and traditional Chinese music.^35^

In only six studies, the music was selected by the patients.^35^^,^^36^^,^^39^^,^^40^^,^^41^^,^^43^ In the other nine studies, the investigators selected the music.

All studies indicate that the intervention was developed in the evening, before bedtime, beginning between 8 and 11 p.m., except Wen & Fan,^36^ whose personalized music therapy protocol included both a morning (10:00 a.m.) and an evening (8:00 p.m.) session daily. The duration of the intervention varies across studies, ranging from 5 min (minimum)^33^ to all night (maximum),^43^ but in most studies, the investigators delivered the intervention for 30 min.^25^^,^^34^^,^^35^^,^^36^^,^^39^^,^^40^

Only two studies refer to the outcome “duration of sleep”,^31^^,^^42^ while all other studies analyzed sleep quality. In the quasi-experimental study by Afshar et al.,^31^ the intervention group exposed to white noise showed an increase in sleep duration relative to the control group, and this gain was sustained across three nights of hospitalization rather than diminishing over time. Similarly, in the randomized controlled trial by Ryu et al.,^42^ sleep duration increased when patients listened to sleep-inducing music (classical/instrumental, relaxation, and nature sounds) combined with eye shields. The relative scarcity of duration-based outcomes in this literature likely reflects the greater measurement burden of tracking sleep duration accurately in hospital settings without continuous objective monitoring (e.g., actigraphy or polysomnography), compared with the brief end-of-night questionnaires used to capture perceived sleep quality; nonetheless, duration is a clinically meaningful indicator—closely tied to recovery and delirium risk—that warrants more consistent reporting in future trials.

### Sleep quality

The most utilized scale for evaluating sleep quality was the Richards-Campbell Sleep Questionnaire (RCSQ).^33^^,^^36^^,^^37^^,^^38^^,^^40^^,^^41^^,^^43^^,^^44^ The RCSQ is a concise tool designed to evaluate sleep quality and alertness. It comprises five items: sleep depth, sleep latency, awakenings, returning to sleep, sleep quality, and noise. Each item is rated on a scale from 0 to 100, with higher scores indicating better sleep quality and alertness.

Following RCSQ, the Pittsburgh Sleep Quality Index Questionnaire (PSQI) was used in one study^31^ as the primary outcome measure; Hu, Jiang, Hegadoren et al.^38^ additionally administered the PSQI at baseline only, to characterize habitual sleep quality and confirm comparability between groups prior to the intervention. The Verran and Synder-Halpern (VSH) Sleep Scale was chosen in two studies.^32^^,^^42^

The PSQI evaluates various dimensions of sleep, including sleep duration, latency, efficiency, disturbances, medication use, and daytime dysfunction. It generates seven component scores, which are then summed to yield a global score ranging from 0 to 21. Higher scores on the PSQI indicate poorer sleep quality.

The VSH and its adapted versions assess dimensions such as sleep latency, duration, disturbances, and overall sleep quality. Because scoring direction varied across the versions used in the included studies, scale directionality was verified at study level and harmonized before quantitative synthesis.

In this review, we explored heterogeneity, using the standardized mean difference (SMD) as an effect measure once the studies utilized different scales to assess sleep quality.^21^ By standardizing mean differences onto a single scale, the SMD allowed the comparison and synthesis of effects found across different studies, providing a more comprehensive and robust understanding of sleep quality (Figure 2). The studies by Herscher et al.^33^ and Kakar et al.^39^ were not included in this analysis as they needed to provide the mean scores of the scales used.

**Figure 2:**
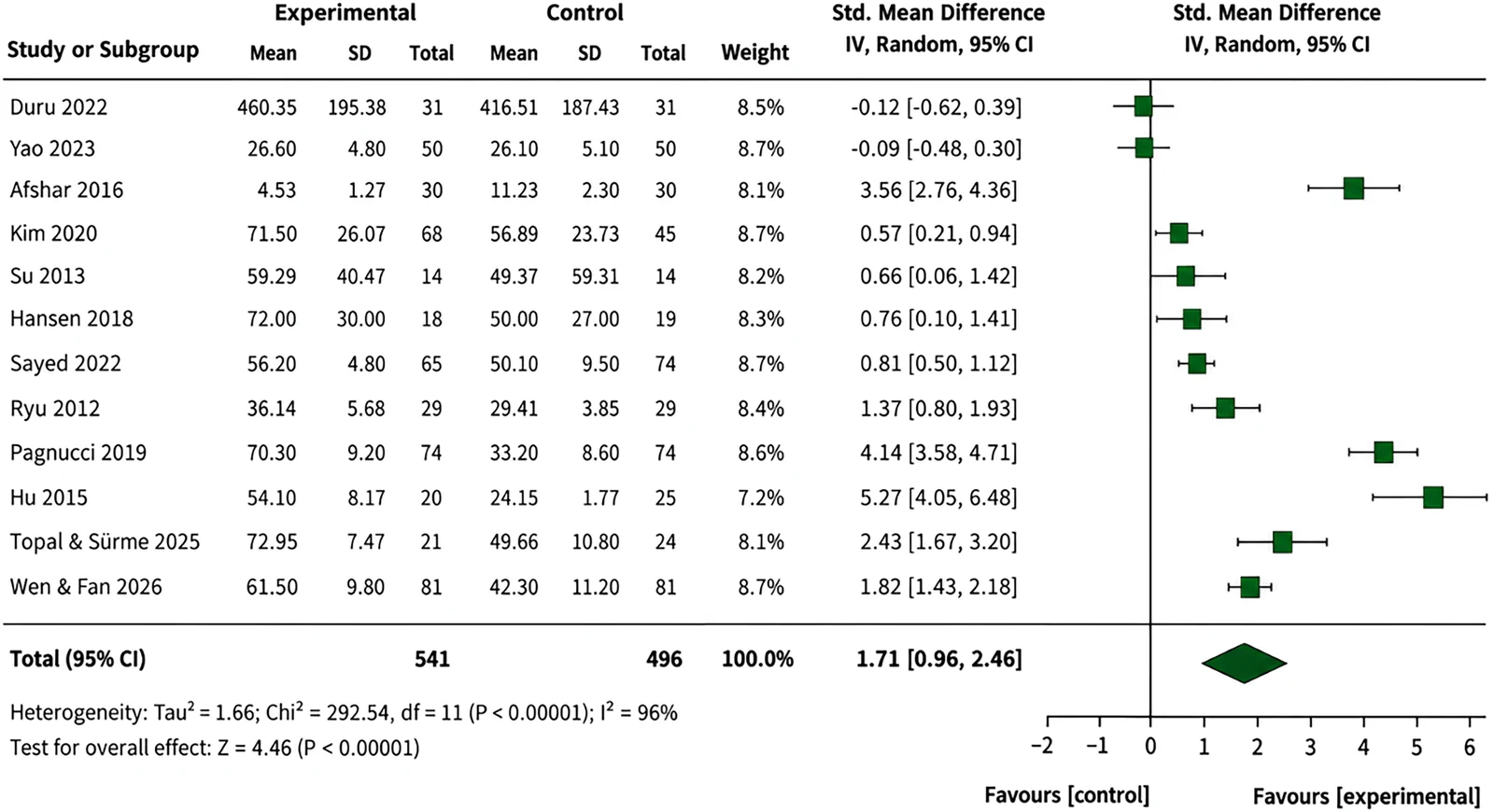
Forest plot of the effects of sound-based interventions on sleep quality (standardized mean difference, random-effects model) “Favors [experimental]” indicates that the sound-based intervention was associated with better sleep quality than the comparison condition; “Favors [control]” indicates the opposite direction of effect. Squares represent the point estimate for each study, with square size proportional to study weight, and horizontal lines represent the 95% confidence interval; the diamond represents the pooled effect estimate.

The heterogeneity observed among the included studies (I^2^ = 96%) was accounted for using a random-effects model (DerSimonian-Laird) to pool the standardized mean difference.^24^ The pooled analysis indicated that sound-based interventions were associated with a significant, large improvement in sleep quality (SMD = 1.71, 95% CI [0.96, 2.46]; Tau^2^ = 1.66; *Z* = 4.46, *p* < 0.00001) (Video S1).

Video S1. Decoding_Figure_2_s_Forest_Plot

A sensitivity analysis excluding the lowest-quality study (Afshar et al.^31^; 44% on JBI appraisal) yielded a somewhat lower but still large and significant pooled estimate (SMD = 1.54, 95% CI [0.79, 2.29] vs. 1.71 [0.96, 2.46] in the primary analysis), indicating that the overall finding was not driven solely by this single study, although its exclusion attenuated the effect size. Chaudhary et al.^25^ was not included in the pooled analysis due to its crossover design lacking an independent control arm and is described narratively later in discussion.

A subgroup analysis by study design showed a large, significant pooled effect among both RCTs (*k* = 8, *N* = 608; SMD = 1.50, 95% CI [0.72, 2.27]; *I*^2^ = 94%) and quasi-experimental studies (*k* = 4, *N* = 440; SMD = 2.08, 95% CI [0.20, 3.96]; *I*^2^ = 98%). While both subgroups indicated a beneficial effect of sound-based interventions, the quasi-experimental subgroup yielded a larger and less precise estimate with greater heterogeneity, consistent with the higher risk of bias associated with non-randomized designs. This pattern supports interpreting the overall pooled estimate with appropriate caution, while indicating that the direction and significance of the effect were not an artifact of pooling RCTs with quasi-experimental studies.

Several factors likely contributed to this substantial heterogeneity. First, the interventions themselves were heterogeneous in nature, encompassing classical music, relaxation music, nature sounds, white noise, and traditional music, delivered for durations ranging from 5 min to an entire night. Second, patient populations differed in acuity and clinical context: most studies were conducted in ICU settings, where sedation, mechanical ventilation, and acute illness severity plausibly modify both baseline sleep disruption and responsiveness to auditory stimuli, whereas three studies were conducted in general wards. Third, the person selecting the sound differed across studies—patients chose the music in six studies, while investigators selected it in the remaining nine—and patient-selected, preference-matched sound may plausibly produce larger effects than investigator-assigned sound, although the available data did not allow us to test this hypothesis formally. Given the small number of studies within each potential subgroup, a formal moderator or subgroup analysis was not feasible; we therefore present these as plausible explanatory hypotheses for the observed heterogeneity (*I*^2^ = 96%), rather than as tested effects, and recommend that future trials report outcomes stratified by intervention type, care setting, and sound-selection method to allow such analyses.

Eight of the twelve pooled comparisons involved sound combined with another sensory, care, or environmental component—massage (Pagnucci et al.^41^: SMD = 4.14, 95% CI [3.57, 4.72]; Sayed et al.^43^: SMD = 0.81, 95% CI [0.49, 1.12]), earplugs and/or an eye mask (Ryu et al.^42^: SMD = 1.37, 95% CI [0.79, 1.94]; Hu et al.^38^: SMD = 5.27, 95% CI [3.99, 6.55]; Topal & Sürme^44^: SMD = 2.43, 95% CI [1.67, 3.20]), a structured relaxation/rehabilitation program (Duru et al.^34^: SMD = −0.12, 95% CI [-0.62, 0.37]; Yao et al.^35^: SMD = −0.09, 95% CI [-0.48, 0.30]), or an environmental noise-control technology (Wen & Fan^36^: SMD = 1.82, 95% CI [1.45, 2.18])—while four comparisons delivered sound alone, with no adjunct component (Afshar et al.^31^: SMD = 3.56, 95% CI [2.76, 4.36]; Kim et al.^40^: SMD = 0.57, 95% CI [0.21, 0.94]; Su et al.^32^: SMD = 0.69, 95% CI [-0.08, 1.45]; Hansen et al.^37^: SMD = 0.76, 95% CI [0.09, 1.43]). Sound-alone comparisons were consistently positive in direction but varied considerably in magnitude (SMD 0.57 to 3.56), a range driven largely by the very large effect observed in Afshar et al.^31^ relative to the more modest effects in Kim et al.^40^, Su et al.^32^, and Hansen et al.^37^ Combined interventions spanned an even wider and less consistent range (SMD -0.12 to 5.27), including two comparisons with negligible or null effects^34^^,^^35^ alongside others showing very large effects^38^^,^^41^; the two newly added combined comparisons^36^^,^^44^ fell within this pre-existing range, reinforcing rather than altering the pattern. This pattern suggests that neither sound-alone nor combined interventions form a homogeneous category, and that factors other than the mere presence of an adjunct component -- such as individual study quality, delivery format, or population—likely contribute to the heterogeneity observed (I^2^ = 96%).

## Discussion

This review examines various sound interventions investigated across the studies, from classical music and relaxation melodies to sounds of nature and white noise. Previous studies have shed light on auditory stimuli’s impact on sleep architecture.^45^^,^^46^^,^^47^ Our findings suggest that sound-based interventions promote sleep quality in hospitalized patients. Similar positive effects of audio stimuli on sleep have been observed in various studies involving different demographic groups. Auditory stimulation exerts an influence not only on the time it takes to fall asleep (latency) but also on the overall duration of sleep, the efficiency of sleep, the period of wakefulness after falling asleep, and the composition of sleep stages (alterations in the proportion of each sleep stage within the total sleep period).^48^^,^^49^

These effects are thought to arise through at least three complementary mechanisms, which likely differ by sound type. First, masking sounds such as white noise are believed to act by raising the auditory arousal threshold: a continuous, non-alerting background sound reduces the acoustic contrast created by intermittent disruptive noises (alarms, conversations, equipment), making the cerebral cortex less likely to register them as arousal-triggering stimuli.^45^^,^^49^ Second, calming or preference-matched music is thought to act primarily through autonomic and neuroendocrine pathways: auditory input projects not only to the auditory cortex but also to limbic structures involved in emotional regulation, promoting parasympathetic activation, reduced sympathetic arousal, and attenuated cortisol and catecholamine release, with consequent reductions in heart rate, blood pressure, and perceived anxiety that favor sleep onset and maintenance.^49^ Third, more targeted approaches such as closed-loop acoustic stimulation, timed to coincide with endogenous slow-wave sleep oscillations, have been shown to directly enhance slow-wave activity, suggesting a more specific neurophysiological pathway distinct from simple masking or relaxation.^46^^,^^47^ These distinct mechanisms may partly explain the heterogeneous effect sizes observed across intervention types in the present review, and future primary studies would benefit from specifying and, where possible, measuring the mechanistic pathway most relevant to the sound-based intervention under investigation.

Sound-based interventions yield improved outcomes when combined with other approaches. Providing sleep packages (eye masks, earplugs, lavender scent pads, and non-caffeinated tea bags) is a low-cost intervention, does not require hiring new staff, and may be integrated into existing hospital workflows.^33^ Earplugs and sleep-inducing music could enhance sleep quality in ICU patients.^42^ Aromatherapy, body massage, calming music, and stress reduction in the ICU have been linked to improved sleep patterns.^43^ Finally, progressive relaxation exercises^34^ or feet and leg massages with sweet almond oil and aromas of lavender and lemon, combined with continuous musical sounds overnight, have shown promising results in enhancing sleep quality and reducing stress perception in ICU patients.^41^ Similarly, combining eye masks with calming music^44^ and combining personalized music with environmental noise-control technology^36^ have also shown improvements in sleep quality, further illustrating the range of adjunct components paired with sound in this literature.

The pattern shown in Table 3, combined interventions spanning a much wider range of effects than sound-alone studies, but in inconsistent directions, more plausibly reflects heterogeneity than a clear synergistic advantage. The only study directly comparing a combined intervention with a sound-only comparator within the same sample, Chaudhary et al.,^25^ did find earplugs-plus-eye-mask more effective than ocean sound alone (SMD = −2.56, 95% CI [-3.25, −1.86] and SMD = −1.62, 95% CI [-2.21, −1.03] across two assessment points), the closest evidence in this dataset to a head-to-head test of this question. Given the small number of studies in each category, these figures should be interpreted descriptively rather than as confirmed evidence of a synergistic effect; larger trials with a common sound-alone comparator arm are needed to test this directly. This pattern is broadly consistent with the wider literature. Kakar and colleagues’ earlier meta-analysis of music-only interventions in critically ill and surgical patients (five studies, 259 patients) reported a pooled SMD of 1.21 (95% CI 0.50–1.91), corresponding to an estimated 27% improvement in subjective sleep quality,^15^ a magnitude comparable to the sound-alone effects observed in our review. More recently, a cluster-randomized trial testing a multicomponent sleep-enhancing protocol without any sound component (delayed care routines, sleep hygiene measures, earplugs, and eye masks) in general medical wards reported a higher median Richards-Campbell Sleep Questionnaire score in the intervention group than in controls (66.6 vs. 55.7, *p* = 0.033).^50^ This trial suggests that the larger effects we observe for combined interventions may reflect the general value of addressing multiple sleep disruptors simultaneously, rather than something specific to pairing sound with another modality.

**Table 3:** Exploratory classification of the 12 pooled comparisons by care setting, intervention type, and music-selection method, alongside individual standardized mean differences (SMD) from the meta-analysis (Figure 2)

Study	Setting	Intervention type	Music selector	SMD [95% CI]
Duru et al.^34^	surgical ward	combined (music + relaxation exercises)	researcher	−0.55 [−1.06, −0.05]
Yao et al.^35^	cardiology ward	combined (music + cardiac rehabilitation)	patient	−0.53 [−0.93, −0.13]
Afshar et al.^31^	ICU	sound alone (white noise)	researcher	3.56 [2.76, 4.36]
Kim et al.^40^	ICU	sound alone (music)	patient	0.57 [0.21, 0.94]
Su et al.^32^	ICU	sound alone (music)	researcher	0.69 [−0.08, 1.45]
Hansen et al.^37^	ICU	sound alone (music)	researcher	0.76 [0.09, 1.43]
Sayed et al.^43^	ICU	combined (music + massage + aromatherapy)	patient	0.81 [0.49, 1.12]
Ryu et al.^42^	ICU	combined (music + earplugs + eye mask)	researcher	1.37 [0.79, 1.94]
Pagnucci et al.^41^	ICU	combined (music + massage)	patient	4.14 [3.57, 4.72]
Hu et al.^38^	ICU	combined (music + earplugs + eye mask)	researcher	5.27 [3.99, 6.55]
Topal & Sürme.^44^	ICU	combined (music + eye mask)	researcher	2.43 [1.67, 3.20]
Wen & Fan.^36^	surgical ward	combined (personalized music + IoT noise control)	patient	1.82 [1.45, 2.18]

Sound-alone and combined interventions both spanned wide and, in the case of combined interventions, inconsistent ranges of effect, suggesting that intervention category alone does not account for the observed heterogeneity (I^2^ = 96%).

Two systematic reviews addressing closely related questions were published after our search date and were therefore not captured by our search strategy. Khandelwal et al.^51^ examined sound-machine (primarily white-noise) interventions specifically in hospitalized adults across seven RCTs (496 patients) but could not pool a meta-analysis owing to methodological heterogeneity, reaching conclusions similar to ours regarding the wide variability in intervention duration and outcome measures. Ding et al.^52^ took a broader population approach, meta-analyzing white-noise interventions across infants, adults, older adults, and critically ill/non-critically ill patients (12 RCTs, 1301 participants) and concluded that effects vary by population, recommending individualized protocols. Our review complements and extends both: unlike Khandelwal et al., we were able to pool a quantitative meta-analysis across a broader range of sound types, not limited to white noise; and unlike Ding et al., we focused specifically on the hospitalized population and additionally examined intervention type (sound-alone vs. combined) and music-selection method as candidate sources of heterogeneity (Table 3), an analysis neither parallel review conducted. Together, this body of recent, independent evidence converges on a consistent overall conclusion: sound-based interventions show a consistent, if heterogeneous, benefit for sleep in hospitalized and critically ill patients, while underscoring the field’s shared need for more standardized outcome measurement.

The diverse range of sound stimuli highlights the need to customize interventions according to individual preferences. In the context of sound-based interventions for promoting sleep, the emphasis on tailoring interventions to individual preferences aligns closely with this patient-centered approach.^53^ Emphasizing the significance of using pleasant sounds for effective intervention, this approach highlights the need for a continuous nurse-patient relationship built on trust for successful personalization.^53^^,^^54^ This approach is consistent with other studies that reinforce the need for individualized approaches in addressing sleep disorders.^5^

Complex healthcare environments are vital for caring for vulnerable patients in critical or acute settings, where excessive sensory stimuli, such as noise and light, can harm patient care within these environments. In these contexts, fundamental nursing care practices, such as promoting restorative sleep, are frequently overlooked.^54^^,^^55^^,^^56^ Our review shows that most of the studies took place in the ICU,^25^^,^^31^^,^^32^^,^^37^^,^^38^^,^^40^^,^^41^^,^^42^^,^^43^ where it is known that the patients have a poor quality of sleep, with less deep sleep stages and rapid eye movement (REM) and with increased arousals.^57^^,^^58^ This is due not only to critical illness but also because of artificial light, increased noise, and healthcare interventions that can disturb sleep.^59^ Patients identified as the most extrinsic disruptor factors of sleep noise, lighting, and nursing interventions.^60^

The World Health Organization recommends that sound levels should not exceed 35 dB in the daytime and 40 dB at night in hospital environments.^13^ However, in a hospital setting, noise levels can vary from 37 to 88.6 dB during the daytime and from 38.7 to 68.8 dB at night.^61^ Also, continuous exposure to artificial light alters circadian sleep and melatonin secretion, which regulates the sleep-wake cycle. This is why ICU design is recommended to have a big window to the outdoors that allows natural light.^60^^,^^62^

Environmental factors are also a risk factor for developing delirium, which will also lead to an abnormal circadian rhythm, and that will affect sleep quality.^60^ Consequently, growing evidence highlights the crucial role of environmental interventions in both preventing and managing delirium.^63^

Nurses have a crucial role in managing healthcare environments to ensure patient safety and prevent issues arising from sleep deprivation.^64^^,^^65^ This aligns with the principles advocated by the International Council of Nurses, which emphasizes the responsibility of nurses to promote a safe and supportive environment for patients.^66^ According to the same source, environmental process and sleep are the focus of nursing care, which involves an integrated patient-centered approach that emphasizes environmental and psychosocial factors as integral components of care, especially in complex settings (e.g., ICUs). This approach requires a reevaluation of current practices to ensure they support the holistic well-being of vulnerable patients, focusing on environment optimization, noise reduction (e.g., decrease or eliminate routine, non-essential nighttime care), lighting adjustment, healing environment promotion, integration of sleep-promotion strategies into daily nursing care plans (e.g., use or instruct the use of electronic devices, such as headphones to listen to music or other therapeutic sounds, according to patients’ preferences), and documentation of instructions and specific responses for patients, recognizing the complex interplay between environmental factors and patients’ outcomes. In the same way, nurses have an essential role in behavior and policy changes, educating themselves, patients, families, healthcare professionals, and organizations about the significance of environmental factors on patients’ outcomes.

In practice, this role can be operationalized through a structured, stepwise pathway that reflects the integration of physical, psychosocial, and relational care dimensions central to the Fundamentals of Care framework^54^: (1) assessing each patient’s sound preferences and any contraindications (e.g., hearing impairment, agitation) at admission or shortly thereafter, as a relational, person-centered assessment rather than a uniformly applied protocol, consistent with McCormack’s^53^ person-centered practice framework; (2) selecting and preparing preferred audio content, delivered via headphones or bedside speakers to minimize disturbance to roommates, addressing the physical care dimension of the intervention; (3) scheduling the intervention within the evening care routine, ideally clustering nursing procedures (e.g., vital signs, medication administration) before the intervention window to reduce interruptions during the following period, in line with evidence that minimizing non-essential nocturnal care disruptions protects patients’ sleep^5^; (4) documenting the intervention delivered, its timing, and the patient’s subjective response in the nursing record, to support relational continuity of care across shifts; and (5) reassessing preferences periodically, as they may change over the course of hospitalization, reflecting the cyclical and ongoing nature of the nursing process rather than a one-off action. At the policy level, standardized guidelines should specify recommended sound types and delivery modes by care setting (e.g., ICU versus general ward), minimum and maximum intervention durations, criteria for excluding patients (e.g., specific psychiatric or neurological conditions), and indicators for monitoring implementation fidelity and patient-reported outcomes, consistent with recent calls for nurse managers to operationalize Fundamentals of Care-based approaches through structured organizational strategies.^56^

By actively engaging in environmental management, nurses uphold Nightingale’s holistic approach to care, valuing ecological control to create therapeutic environments. This contributes to patient recovery and well-being, thereby enhancing patients’ chances of recovery through the cultivation of a healing-conducive environment.^11^ Nightingale’s advocacy highlights nurses’ pivotal influence in shaping the physical and emotional spaces where patients receive care.

Future research should, therefore, prioritize randomized controlled trials with larger sample sizes and studies in which sound interventions are the sole focus, studied individually for each type of sound, without the confounding influence of multimodal interventions that simultaneously include other strategies (e.g., massage, aromatherapy or the concomitant use of different sounds), to prove the effect of each sound on patients’ sleep.^25^^,^^33^^,^^38^^,^^41^^,^^42^^,^^43^^,^^67^ A challenge described in the evidence may be to demonstrate the effectiveness of these sounds individually. At the same time, nurses must limit interventions that cause sleep disturbance and bias in the study by managing the environment and timing of nursing care activities.^5^

### Limitations of the study

The current review had limitations in study quality that compromised the internal validity and reliability of these studies. Significant methodological heterogeneity was observed among the included studies, particularly regarding the type, timing, and duration of sound-based interventions, as well as the instruments used to measure sleep quality. This instrument heterogeneity was problematic in three specific ways. First, the included instruments and their adapted versions differed in scale directionality. Higher RCSQ scores indicate better sleep quality, whereas higher PSQI and SRSS scores indicate poorer sleep quality; VSH-derived measures and adapted sleep scales varied in scoring direction across studies. Reconciling these opposing directions required careful reverse-scoring during data extraction to ensure a consistent interpretation of the pooled effect (see data synthesis); an error at this step would silently invert the direction of a study’s contribution to the pooled estimate, and this risk is likely under-recognized in meta-analyses of sleep-quality interventions more broadly. Second, the instruments differ in the construct and recall period assessed: the RCSQ, VSH, and SRSS capture perceived sleep over the preceding night, whereas the PSQI, as originally validated, assesses habitual sleep quality over the preceding month; using the PSQI to capture the effect of a single-night or short-duration intervention represents a construct mismatch that may attenuate or distort observed effects. Third, validation status varied considerably: while the RCSQ has been validated against polysomnography in ICU populations, other instruments were adapted or developed ad hoc for a single study without independent psychometric testing (e.g., Kakar et al.^39^; Chaudhary et al.^25^), raising uncertainty about their reliability and construct validity. None of the included studies employed an objective measure of sleep (e.g., actigraphy or polysomnography) as the primary outcome. Future primary studies should prioritize validated instruments with an appropriate recall period matched to the intervention duration, explicitly report scale directionality to facilitate accurate data extraction in future reviews, and, where feasible, supplement subjective self-report with objective measures such as actigraphy. Future reviews and meta-analyses in this field should explicitly verify and document scale directionality for each included instrument as a standard data-extraction step, given the risk of silent sign errors when pooling heterogeneous sleep-quality measures.

The largest individual effect size in the pooled analysis^31^ was contributed by the lowest-quality study in the sample (44% on JBI appraisal), warranting caution in interpreting its magnitude; however, a sensitivity analysis excluding this study did not materially change the overall pooled estimate. Additionally, the inclusion of both pre-recorded and live/interactive music delivery formats (e.g., Kim et al.^40^) introduces a further source of clinical heterogeneity that may partly explain the high I^2^ observed.

It is important to note that study design (RCT versus quasi-experimental) does not map directly onto methodological quality in this sample: the highest JBI appraisal score (100%, Pagnucci et al.^41^) was achieved by a quasi-experimental study, exceeding that of any included RCT (maximum 77%), while the lowest score (44%, Afshar et al.^31^) was also quasi-experimental. This underscores that design type alone is an imperfect proxy for risk of bias, and supports our decision to assess and report study-level quality (Table 2) rather than relying solely on the RCT/quasi-experimental distinction to judge evidentiary weight.

A related limitation concerns the substantial clinical heterogeneity of the patient populations sampled across the included studies. The sample spanned stable postoperative elective surgical patients (Kim et al.^40^; Duru et al.^34^ across general, urological, plastic, orthopedic, cardiovascular, ophthalmologic, neurologic, thoracic, and ENT surgical specialties), patients undergoing abdominal or perianal surgery,^36^^,^^44^ general medical ward patients,^33^ and critically ill patients in intensive or coronary care units with heterogeneous underlying diagnoses (e.g., Chaudhary et al.,^25^ whose ICU sample included gastrointestinal, respiratory, cardiovascular, neurological, urinary, and endocrine admissions), alongside populations selected for specific cardiac diagnoses or procedures (acute myocardial infarction, percutaneous coronary intervention, coronary artery bypass grafting, unstable angina). Disease type, severity, and concurrent treatments (e.g., sedation, analgesia, mechanical ventilation) are established, independent determinants of sleep quality in hospitalized patients and may confound the observed association between sound-based interventions and sleep outcomes. Most included studies assessed baseline comparability between groups using simple univariate tests rather than multivariable adjustment for illness severity or treatment burden, limiting our ability to disentangle the effect of the sound-based intervention from that of these clinical confounders. Given the limited number of studies per population subgroup, we were unable to conduct a formal subgroup analysis stratified by disease type or severity; future primary studies should report and, where feasible, statistically adjust for severity-of-illness indices (e.g., APACHE II) and treatment-related sleep disruptors, and future reviews with a larger evidence base should examine population type as a potential moderator of intervention effectiveness.

#### Implications for practice

This review highlights the potential of sound-based interventions to improve sleep quality in hospitalized patients, a crucial aspect of recovery and overall well-being. In clinical practice, nurses can incorporate therapeutic sounds, such as calming music or nature sounds, into care routines to foster a restful environment. By reducing everyday hospital stressors such as noise and light disruptions, nurses actively create a supportive space conducive to quality sleep.

Creating sleep-promoting environments fulfills an essential need that significantly impacts patients’ mental health and emotional resilience. Therapeutic sounds can help reduce anxiety, alleviate stress, and promote relaxation, providing patients with relief from the psychological demands of hospitalization. For patients facing heightened mental distress, these interventions offer grounding effects that aid in emotional regulation and foster a sense of calm.

Standardizing sound-based interventions can contribute to a holistic approach to healthcare delivery. By personalizing these strategies, nurses promote mental health, supporting recovery and well-being at multiple levels. This positions nurses as both caregivers and advocates for mental health, with the ability to create therapeutic environments that bolster patients’ mental resilience.

Clear guidelines and policy initiatives would support nurses in adopting sound-based interventions, further validating, and enhancing their therapeutic impact. Such policies could facilitate the consistent integration of sound therapies in mental health settings, aligning with holistic, recovery-focused mental health care goals.

Expanding nursing education to include training in sound-based interventions would further strengthen these practices, equipping nurses to use these techniques effectively for mental health promotion. Emphasizing the connection between sleep, sound, and mental health in training would empower nurses to use sound therapy confidently, reinforcing the profession’s commitment to mental health advocacy and innovative, evidence-based care approaches.

### Conclusions

Sound-based interventions have emerged as a promising strategy for improving sleep quality among hospitalized patients, particularly when tailored to individual preferences. Various sounds, including classical music, relaxation melodies, and nature sounds, demonstrate the potential to promote better sleep. Moreover, combined approaches have shown promise in further enhancing sleep quality for these patients.

Nurses are crucial in managing healthcare environments to ensure patient safety and address sleep-related issues. By actively engaging in environmental management, nurses uphold Florence Nightingale’s holistic approach to care, contributing to patient recovery and well-being. This underscores nurses’ pivotal influence in shaping therapeutic environments and promoting patient health.

Methodological shortcomings, including diverse outcome measures and a need for standardized assessment tools, contributed to substantial heterogeneity (*I*^2^ = 96%) that constrains confidence in the pooled meta-analytic estimate despite the significant overall effect observed. Future studies should prioritize well-designed randomized controlled trials with larger sample sizes and standardized outcome measures to address these gaps. Such studies will generate more robust evidence on the effectiveness of sound-based interventions in enhancing sleep quality among hospitalized patients.

#### What is already known

• Sleep quality among hospitalized patients is frequently impaired by environmental disturbances, particularly excessive noise and artificial light.
• Non-pharmacological strategies, including sound-based interventions, have shown promising effects in enhancing sleep quality without adverse effects.
• The existing evidence is fragmented, lacking consensus on the most effective types of sound interventions, their optimal timing, duration, and contextual application in clinical settings.

#### What this paper adds

• This systematic review consolidates findings from 15 studies exploring the impact of sound-based interventions on sleep quality in hospitalized patients, with a focus on intensive care settings.
• It highlights classical music, relaxation melodies, and nature sounds as the most commonly applied and potentially beneficial auditory stimuli for sleep promotion.
• The review underscores the importance of individualized, nurse-led interventions tailored to patient preferences and integrated within broader environmental management strategies, calling for standardized guidelines and further research.

## Resource availability

### Lead contact

Further information and requests should be directed to and will be fulfilled by the lead contact, Helga Rafael Henriques (hrafael@enfermagem.ulisboa.pt).

### Materials availability

This systematic review did not generate new physical materials, reagents, or biological samples.

### Data and code availability

The standardized data-extraction spreadsheet and the full search strategies for all five databases generated during this review are available from the lead contact upon request. No original statistical code was written for this review; the software used to generate the meta-analysis and forest plot (Figure 2) is identified in the key resources table above. This paper does not report standardized datatypes requiring deposition in a community repository.

## Acknowledgments

The authors thank the institutional librarian for assistance in retrieving full-text articles during the review process. This research received no specific grant from any funding agency in the public, commercial, or not-for-profit sectors.

## Author contributions

Conceptualization, H.R.H.; methodology, H.R.H. and J.T.; investigation (search strategy, screening, and data extraction), H.R.H., J.T., R.G., M.S., S.C.M., C.D., and M.R.P; formal analysis, H.R.H., J.T.; writing – original draft, H.R.H.; writing – review and editing, H.R.H., J.T., R.G., M.S., S.C.M., C.D., and M.R.P.; supervision, H.R.H. and J.T.; funding acquisition, not applicable.

## Declaration of interests

The authors declare no competing interests.

## Declaration of generative AI and AI-assisted technologies in the manuscript preparation process

During the preparation of this work, the authors used Claude (Anthropic) to assist with verification of extracted data against primary source articles, drafting and editing of manuscript text. After using this tool, the authors reviewed, verified, and edited all content as needed and take full responsibility for the content of the publication.

## STAR★Methods

### Key resources table

**Table:** 

REAGENT or RESOURCE	SOURCE	IDENTIFIER
**Deposited data**		

Systematic review protocol (this study)	PROSPERO	CRD42024511972

**Software and algorithms**		

Rayyan QCRI (reference screening and deduplication)	Rayyan Systems Inc.	https://www.rayyan.ai/
RevMan (Review Manager), version 5.4	The Cochrane Collaboration	https://revman.cochrane.org/

**Other**		

JBI Critical Appraisal Checklist (RCTs and quasi-experimental studies)	Joanna Briggs Institute	https://jbi.global/critical-appraisal-tools
PRISMA 2020 reporting guideline	Page et al., 2021	https://doi.org/10.1136/bmj.n71
SWiM (Synthesis without Meta-analysis) reporting guideline	Campbell et al., 2020	https://doi.org/10.1136/bmj.l6890

### Experimental model and study participant details

This systematic review synthesized data from 15 published studies involving adult patients hospitalized overnight in intensive care, coronary care, or general medical/surgical wards (see eligibility criteria in methods). No new human participants were recruited for this review; all participant-level data derive from the original published studies, each of which reported institutional ethics approval and informed consent procedures as described in their respective publications. Across the included studies, sex/gender distribution was reported inconsistently and, where reported, samples included both male and female participants in varying proportions (e.g., Chaudhary et al.^25^: 51.7% male; Duru et al.^34^: approximately 65% male in both groups). None of the included studies formally examined sex or gender as a moderator of intervention effectiveness, and this review was therefore unable to assess whether sound-based interventions differ in effect by sex or gender. This represents a limitation of the current evidence base and is noted as a direction for future primary research.

### Method details

See search strategy, Outcome, selection process, Data collection process and data items, and study risk of bias assessment in the methods section above.

### Quantification and statistical analysis

See data synthesis in the methods section above.

### Additional resources

Systematic review protocol: PROSPERO registration CRD42024511972. https://www.crd.york.ac.uk/prospero/display_record.php?ID=CRD42024511972.
